# Molecular characterization of ochratoxigenic fungi associated with poultry feedstuffs in Saudi Arabia

**DOI:** 10.1002/fsn3.1827

**Published:** 2020-08-19

**Authors:** Youssuf A. Gherbawy, Hesham M. Elhariry, Saad A. Alamri, Eman G.A. El‐Dawy

**Affiliations:** ^1^ Applied and Environmental Microbiology Center South Valley University Qena Egypt; ^2^ Botany and Microbiology Department Faculty of Science South Valley University Qena Egypt; ^3^ Department of Food Science Faculty of Agriculture Ain Shams University Cairo Egypt; ^4^ Biology Department Faculty of Science King Khalid University Abha Saudi Arabia; ^5^ Research Center for Advanced Materials Science (RCAMS) King Khalid University Abha Saudi Arabia

**Keywords:** *Aspergillus*, ochratoxin A, *Penicillium*, genes, Poultry

## Abstract

Fungal and mycotoxins contamination of food and poultry feeds can occur at each step along the chain from grain production, storage, and processing. A total of 200 samples comprising of mixed poultry feedstuffs (*n *= 100) and their ingredients (*n = *100) were collected from Riyadh, Alhassa, Qassium, and Jeddah cities in Saudi Arabia. These samples were screened for contamination by fungi. *Penicillium chrysogenum* was the predominant species taking into its account and frequency, respectively, in both mixed poultry feedstuff and barley samples (4,561.9 and 687 fungal colony‐forming units (CFU)/g) and (66% and 17%). Moisture content was an important indicator for the count of fungi and ochratoxin A. Ochratoxin analysis of plate cultures was performed by a HPLC technique. Sample of mixed poultry feedstuff which was collected from Jeddah displayed the highest level of ochratoxin (14.8 µg/kg) and moisture content (11.5%). Corn grains samples were highly contaminated by ochratoxin A (450 and 423 µg/kg) and recorded the highest moisture contents (14.1 and 14.5%). Ochratoxin A production in fungal species isolated from mixed poultry feedstuff samples were high with *P*.* verrucosum* (5.5 μg/kg) and *A*.* niger* (1.1 μg/kg). In sorghum and corn grains, the highest ochratoxins producing species were *P*.* viridicatum* (5.9 μg/kg) and *A*.* niger* (1.3 μg/kg), respectively. Sixty‐three isolates of *A*.* niger* were ochratoxigenic, and all of them showed the presence of *pks* genes using PKS15C‐MeT and PKS15KS primer pairs. The detection technique of *A*.* niger* in poultry feedstuff samples described in the present study was successfully used as a rapid and specific protocol for early detection of *A*.* niger* without cultivation on specific media.

## Graphical abstract

This study reported the identification of fungal species that contaminated poultry feed‐stuff samples during storage, detection of ochratoxins A and also evaluating a rapid technique for testing fungal contamination in poultry feed‐stuff without culturing of the fungi.

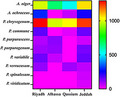

## 1 INTRODUCTION

Agricultural products including oilseed meals and cereals constitute a major component of poultry feed ingredients. Fungal contamination is widely distributed in tropical countries, where poultry production and processing are extending quickly (Okoli, Nweke, Okoli, & Opara, 2006; Vanden & Ahouanginou, 1990).

The microbial diversity found in various feeds is dependent upon the water activity, pH, oxygen tension, and nutrient composition of the feed material. Preserving feedstuffs ingredients and feeds is of great interest to the poultry industry and feeding. Water activity (a_w_) and temperature are the most critical environmental factors (Charmley & Prelusky, 1994). Several studies have been done to determine *Aspergillus* spp. growth and mycotoxin production, on irradiated grains (Cuero, Smith, & Lacey, 1987), on poultry feed (Dalcero, Magnoli, Chiacchiera, & Palacio, 1997), barley and wheat grains (Baliukoniene & Bakutis, 2003), and mixed feeds (Accensi, Abarca, & Cabañes, 2004). Little attention has been paid to the effect of incubation temperature, changes in a_w,_ and incubation time on mycotoxin production by *Aspergillus* spp. (Ribeiro, Wenseleers, Santos Filho, & Alves, 2006).

In *A*.* ochraceus*, as shown for other mycotoxins (Northolt & Bullerman, 1982) the range of *a*_w_ × temperature conditions conducive to growth was much wider than that for ochratoxin A (OTA) production. Growth occurred over the temperature range 8–37ºC **(**ICMSF, 1996 **)** with an optimum of about 30ºC on barley grains (Ramos et al., 1998). The maximum contents of OTA were achieved at 0.98 *a*_w_, regardless of the temperature level, with maximum OTA production optimum between 25–30ºC, depending on the strains. Referring to *a*_w_, both growth and OTA potential increased by increasing *a*_w_ levels until 0.96–0.98, with 0.80 as the minimum *a*_w_ for growth on maize‐based media (Marín, Ramos, & Magan, 1998) and 0.83–0.87 as the minimum *a*_w_ for OTA production (Northol, Van Egmond, & Paulsch, 1979). The matrix of food and nutritional status are very significant in detecting of OTA production (Madhyastha, Marquardt, Frohlich, Platford, & Abramson, 1990).

The first report of ochratoxin A production in barley with *Eurotium* spp. in Saudi Arabia has been published by Al‐Julaifi (2003). He stated that three isolates of *Eurotium* (*E*.* amstelodami*, *E*.* herbariorum,* and *Eurotium* spp) have been found to produce ochratoxin A with different concentrations. Quantities of ochratoxin A were detected in YES by *E*.* amstelodami, E*.* herbariorum,* and *Eurotium* spp., and they produced 138.21, 302.17, and 245.25 mg/kg, respectively. In PDA, only *E*.* amsielodami* and *Eurotiun* spp were able to produce ochratoxin A at concentrations of 97.14 and 205.13 mg/kg, respectively. Ochratoxin A production on barley medium were 165.14, 121.39, and 214.77 mg/kg with *Eurotium* spp, *E*.* amstelodami,* and *E*.* herbariorum*, respectively.

The traditional schemes for the isolation and identification of ochratoxigenic fungi from food samples are time‐consuming and require a high knowledge of fungal taxonomy. Even with taxonomic expertise, identification is commonly difficult in some genera of fungi that contain a large number of closely related species. *Aspergillus* Section *Nigri* includes several species difficult to be identified by traditional methods because differences are mainly based on the uniseriate and biseriate condition of the sterigmata of conidial heads and the size, and roughness of the conidia (Battilani, Barbano, & Piva, 2008). Hence, it is imperative to develop methodologies that are relatively rapid, highly specific, and as an alternative to the existing methods. The application of molecular biology techniques can help to overcome these problems because it can reduce the time for identification from days to hours and also allow precise species identification (Sartori, Bastos, & Wiltbank, 2010). PCR‐based methods that target DNA are considered a good alternative for rapid diagnosis because of their high specificity and sensitivity (Perrone, Susca, Stea, & Mulè, 2004), especially when multi‐copy sequences are used to develop species‐specific primers (Bluhm, Flaherty, Cousin, & Woloshuk, 2002). Patiño, González‐Salgado, González‐Jaén, and Vázquez (2005) developed two PCR assays to detect *Aspergillus carbonarius* and *A*.* ochraceus*, considered as the main sources of ochratoxin A (OTA) contaminating commodities, particularly grapes, coffee, and derivatives, in warm climates. Random amplified polymorphic DNA (RAPD) or amplified fragment length polymorphism (AFLP) have been applied successfully for revealing specific marker sequences, such sequences have been used to design species‐specific primers that allow the identification and detection of some ochratoxigenic species in food samples (Sartori et al., 2006).

Therefore, this work aimed to determine the occurrence and load of fungi, the important foodborne pathogens in mixed feedstuff samples and their ingredients collected from Saudi Arabia, analysis specifically the occurrence of black aspergilli group in these samples, investigation the occurrence of ochratoxin A with chromatographically and molecularly techniques, and direct test of *A*.* niger* in the samples of feedstuff without using cultured media as a fast method in detection of the fungus.

## 2 MATERIALS AND METHODS

### 2.1 Sample collection and handling strategy

Two hundred samples (100 of mixed poultry feedstuff ready for feeding and 100 feedstuff ingredients) were collected from different feed factories, storehouses, and fodder markets in Saudi Arabia. Samples (each about 5 kg) were collected from different regions in Saudi Arabia (Riyadh, Alhassa, Qassium, and Jeddah). Primary samples were homogenized, milled, and quartered to get 1 kg laboratory samples. Proximate microbiological quality and identification of natural mycobiota were done after the day of collection. Another portion of samples stored at −20°C for further work (Gashgari, Shebany, & Gherbawy, 2011).

### 2.2 Moisture content

The moisture content of the collected samples was determined by drying a sample in an oven at a temperature above the boiling point of water (100–105°C) to reach a constant weight (the loss in weight is calculated as a percent of moisture content) (Horwitz & Latimer, 2007).

### 2.3 Enumeration, isolation, and identification of fungi

Quantitative enumeration of fungal colony‐forming units (CFU) was done on solid media using the surface‐spread method (Bokhari, 2010). Twenty grams of ground sample was soaked in 100ml sterile saline water (9 g/L) containing 0.02% Tween 80 and shacked 30 min. From serial dilutions (from 10^–1^ to 10^–5^), one hundred µl aliquots inoculated onto three plates of Dichloran–Rose Bengal–Chloramphenicol Agar (DRBC) medium. Plates were then incubated at 28°C for 7 days. Plates with 10–100 CFU were used for enumeration, and the results expressed as CFU per gram of sample. However, in samples with a low level of fungal contamination, and so plates with less than 10 CFU at the lowest tested dilution (10^–1^) were recorded.

Pure cultures were obtained by transferring hyphal tips to Malt Extract Agar (MEA) with penicillin G and chloramphenicol (MEApc, 75 mg/L MEA). Isolates were maintained on MEA pc at 4°C and identified by macroscopic and microscopic observations. For identification of isolated fungi, the books of Raper and Fennell (1965), Samson (1979), and Pitt and Hocking (2009) were used.

### 2.4 Molecular identification of fungal isolates

#### 2.4.1 DNA isolation

Two ml of potato dextrose broth (PDB) was poured into Eppendorf tubes, inoculated by spores and vortexed to disperse the spores, and the PDB mix was transferred into flasks containing 100 ml of PDB. Flasks were incubated at room temperature without shaking for 2 to 3 days. After filtration, the mycelium was freezed at − 80°C for 30 min, lyophilized, and stored at − 80°C. The lyophilized mycelium was ground in liquid nitrogen in a sterile mortar to obtain a mycelium powder. The DNA was extracted from 20 mg of mycelium powder using DNeasy plant mini kit. The DNA quantity and quality were checked by electrophoresis on a 0.8% agarose gel, revealed with ethidium bromide and visualized by UV trans‐illumination (Gherbawy & Gashgari, 2013).

##### 2.4.2 ITS region sequencing

The internal transcribed spacer (ITS) region of the ribosomal DNA (rDNA) was amplified by PCR primers; ITS1‐*F* (CTTGGTCATTTAGAGGAAGTAA) and ITS4 (TCCTCCGCTTATTGATATGC) (White, Bruns, Lee, & Taylor, 1990). PCR amplifications were done in a final volume of 50 μl by mixing 2 μl of DNA with 0.5 μM of each primer, 150 μM of dNTP, 1 U of Taq DNA polymerase (Promega), and PCR reaction buffer. Amplification was conducted in a thermal cycler by an initial denaturation of 3 min at 94°C, followed by 35 cycles of 1 min at 94°C, 1 min at 50°C, 1 min at 72°C, and a final extension of 10 min at 72°C. Aliquots of PCR products were checked by electrophoresis on a 1% agarose gel revealed with ethidium bromide and visualized by UV trans‐illumination. The PCR products were purified by ExoSAP‐IT (USB Corporation, under license from GE Healthcare) based on the manufacturer's instructions. The purified products were sequenced by using an automated DNA sequencer (ABI PRISM 3700) using the BigDye Deoxy Terminator cycle‐sequencing kit (Applied Biosystems) following the manufacturer's instructions. Sequences were submitted to GenBank on the NCBI website (http://www.ncbi.nlm.nih.gov). The obtained sequences in this study were deposited in GenBank with accession numbers indicated in the Table 3.

#### 2.4.2 Detection and quantification of ochratoxin A

The collected isolates belonging to *Aspergillus niger* and *A*.* ochraceus* group and some of *Penicillium* species were tested for OTA production. OTA was assayed following the methodology described by Téren, Varga, Hamari, Rinyu, and Kevei (1996), with some modifications as follows: the isolates were grown in stationary cultures in 25 ml quantities of YES medium (2% yeast extract, 15% sucrose) at 28°C for 10 days in the dark (Moslem, Mashraqi, Abd‐Elsalam, Bahkali, & Elnagaer, 2010). After incubation, a portion of these culture media (1 ml) was mixed by 1 ml chloroform and centrifuged at 4,000 g for 10 min. The chloroform phase was transferred to a clean tube, evaporated to dryness, and redissolved in 0.5 ml methanol. OTA production was determined by HPLC.

### 2.5 Molecular detection of ochratoxigenic fungi

#### 2.5.1 DNA isolation from *A. niger*

The isolation of DNA from mycelium was performed according to the method described by (Ferracin et al., 2012). Briefly, mycelium from liquid cultures was recovered by filtration and pulverized to a fine powder under liquid nitrogen in a mortar. Approximately 400 mg of the ground mycelium was suspended in 800 ml of lysis buffer (200 mM Tris‐HCl; 250 mM NaCl; 25 mM EDTA; 1% wv‐1 SDS) and maintained at 65°C for 20 min. The DNA was purified with phenol: chloroform (25:24) and chloroform: isoamyl alcohol (24:1) and precipitated in 3M NaCl solution in the presence of 95% ethanol, then washed by 70% ethanol and resuspended in ultrapure water.

#### 2.5.2 Survey of *pks* genes (ochratoxin biosynthetic genes)

Two primer pairs PKS15KS F‐CAATGCCGTCCAACCGTATG, R‐CCTTCGCCTCGCCCGTAG, and PK51C‐Met F‐GCTTTCATGGACTGGATG, R‐CATTTCGTTGATCCCATCG were used to detect the presence of the two different domains of the same gene (An15g07920) that indicated the ochratoxigenic potentials of strains. The reaction conditions were previously described by Ferracin et al. (2012).

#### 2.5.3 Molecular detection of *A. niger* in mixed poultry feedstuff samples

Fungal DNA was isolated from mixed poultry feedstuff ready for feeding samples by enrichment technique. One gram of the sample was cultured in Erlenmeyer flask containing 50 ml of potato dextrose broth tubes (PDB), which was incubated at 30°C for 24 hr in an orbital shaker (140 rpm). DNA extraction was carried out starting from 200 mg of a filtered culture frozen with liquid nitrogen and ground using a mortar and a pestle. All extractions were carried out in triplicate (Gherbawy, Yassmin, & Alharthy, 2016). Elution was carried out in one step by adding 100 μL of elution buffer (TE).

PCR assays were carried out by using primer ITS1 (5′‐TCCGTAGGTGAACCTGCGG‐3′) in all cases combined with a species‐specific primer: NIG (5′‐ CCGGAGAGAGGGGACG GC‐3′) for *A*.* niger*. The PCR amplification protocol used for *A*.* niger* was as follows: 1 cycle of 4 min 30 s at 95°C, 25 cycles of 30 s at 95°C (denaturalization), 25 s at 66°C (annealing), 40 s at 72°C (extension), and finally 1 cycle of 5 min at 72°C. Amplification reactions were carried out in volumes of 25 μl containing 3 μl of template DNA, 1.25 μl of each primer (20 μl), 2.5 μl of 10· PCR buffer, 1 μl of MgCl2 (50 mM), 0.25 μl of dNTPs (100 mM) and 0.2 μl of Taq DNA polymerase (5 U/μl). PCR products were separated in 2% agarose ethidium bromide gels in 1 X TAE buffer (Tris‐acetate 40 mM and EDTA 1.0 mM). The DNA ladder 100 bp was used as a molecular size marker and the positive sample for *A*.* niger* showed 420 bp PCR product (González‐Salgado, González‐Jaén, Vázquez, & Patiño, 2008).

## 3 RESULTS

### 3.1 Mycobiota of various mixed and ingredients of poultry feedstuff samples

The recovered fungal species were identified by microscopic examination, morphological keys, and the identification of fungi was confirmed by using molecular methods. Internal transcribed spacer regions (ITS) of rRNA for representative species of the collected fungal species were sequenced, and the obtained sequence results were deposited in the Gene Bank under accession numbers as shown in Table 3. The sequence results indicated that the molecular identification of the isolated fungi was confirmed the morphological identification.

**Figure 1:**
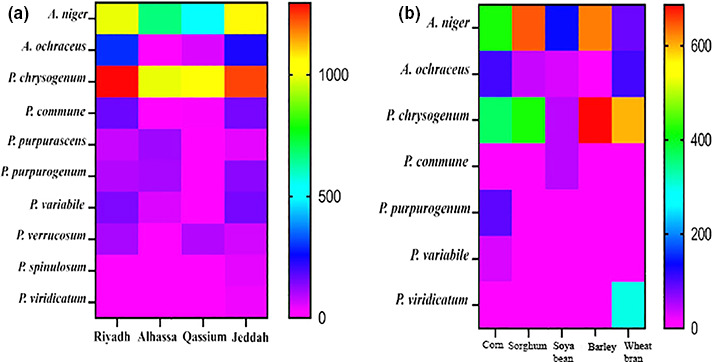
Heatmap of CFU, calculated per g dry feedstuff sample of fungal species were isolated from samples of mixed (a) and ingredients (b) of poultry feedstuff (*n* = 100 of each), which were collected from Riyadh, Alhassa, Qassium, and Jeddah cities on DRBC media at 27°C

**Figure 2:**
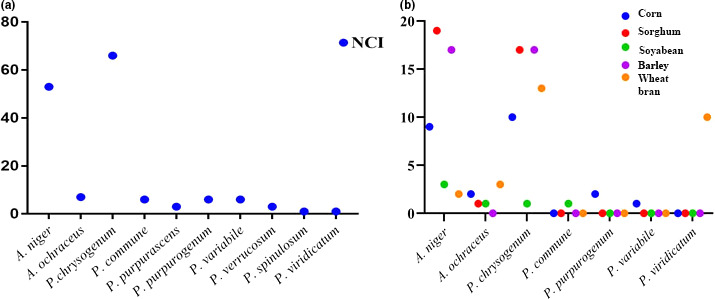
Number of cases of isolation (NCI) of fungal species which were isolated from samples of mixed (a) and ingredients (b) poultry feedstuff (*n* = 100 of each) were gathered from Riyadh, Alhassa, Qassium, and Jeddah cities on DRBC media at 27°C

The samples collected from Jeddah were more contaminated than other regions; 3,181 mean total CFU/g mixed poultry feedstuff and barley samples were more contaminated than the other ingredients samples (1,314 CFU/g). Two genera comprised of a total of 10 species were recovered from 100 samples of mixed poultry feedstuff which were gathered from four regions in Saudi Arabia. Also, two genera involved 7 species were isolated from ingredients of poultry feedstuff samples. *Penicillium chrysogenum* was the predominant species taking into its account and frequency in both mixed poultry feedstuff and barley samples, respectively (4,561.9 and 687 CFU/g) and (66% and 17%). *A*.* niger* ranked the second position in the account and frequency of mixed poultry feedstuff and sorghum samples (3,257.2 and 647 CFU/g) and (53% and 19%), respectively. (Figures 1 and 2).

Moisture content was a considerable factor for the account of isolated fungi. The highest moisture content of the mixed poultry feedstuff sample was marketed from Jeddah city (11.5%). (Table 1).

**Table 1:** The moisture contents (%) and ochratoxin A (µg/kg) of 100 positive mixed poultry feedstuff samples out of 100 samples collected from Riyadh, Alhassa, Qassium, and Jeddah areas

Sample No.	Location	M.C.	OTA	Sample No.	Location	M.C.	OTA
1	Riyadh	9.5	14.5	53	Qassium	6.8	12.6
2	Riyadh	10.2	13.7	55	Qassium	9.6	13.4
4	Riyadh	9.4	12.1	57	Qassium	10.2	14.6
6	Riyadh	7.5	14.3	59	Qassium	9.5	13.2
10	Riyadh	7.2	14.1	62	Qassium	6.4	11.2
12	Riyadh	6.8	14.4	64	Qassium	8.6	13.5
15	Riyadh	6.5	13.2	66	Qassium	10.2	14.1
16	Riyadh	5.4	14.6	67	Qassium	8.5	12.5
18	Riyadh	9.4	14.3	69	Qassium	6.6	11.9
20	Riyadh	10.5	13.5	71	Qassium	6.4	11.3
22	Riyadh	9.4	11.4	73	Qassium	5.6	11.7
23	Riyadh	10.4	14.4	75	Jeddah	6.3	10.5
24	Riyadh	8.5	11.2	76	Jeddah	6.5	11.2
26	Alhassa	6.4	12.3	77	Jeddah	7.4	9.8
28	Alhassa	8.5	13.4	78	Jeddah	6.2	11.3
30	Alhassa	7.3	11.9	80	Jeddah	8.5	13.5
32	Alhassa	6.6	11.7	82	Jeddah	8.6	11.7
33	Alhassa	6.4	12.5	83	Jeddah	6.4	12.5
34	Alhassa	8.5	14.7	84	Jeddah	6.6	14.7
35	Alhassa	6.2	11.2	85	Jeddah	9.4	11.2
36	Alhassa	9.5	13.5	86	Jeddah	10.5	14.1
38	Alhassa	8.4	13.2	88	Jeddah	6.6	10.6
39	Alhassa	7.2	11.2	89	Jeddah	8.6	11.2
41	Alhassa	6.8	11.6	91	Jeddah	8.5	12.3
42	Alhassa	5.3	9.4	93	Jeddah	6.6	8.9
44	Alhassa	10.4	14.1	94	Jeddah	7.5	10.5
46	Alhassa	9.7	13.7	95	Jeddah	7.4	6.8
48	Alhassa	6.5	9.5	96	Jeddah	6.5	9.5
50	Alhassa	7.3	11.8	97	Jeddah	10.4	14.6
51	Qassium	8.2	12.5	99	Jeddah	6.3	8.9
52	Qassium	7.3	11.3	100	Jeddah	11.5	14.8

Note

M.C., Moisture content; OTA, Ochratoxin A.

### 3.2 Naturally occurring ochratoxin A in mixed and ingredients poultry feedstuff samples

One hundred percentage of mixed poultry feedstuff samples were contaminated by ochratoxin (OTA). OTA was recorded from 100 samples of mixed poultry feedstuff samples. Also, the achieved results showed that samples of mixed poultry feedstuff No. 95 and No. 100 which were collected from Jeddah appeared the lowest and highest level of total ochratoxin (6.8 to 14.8 µg/kg), respectively. Seven samples (7%) were contaminated by OTA ranged from 6.8 to 9.8 µg/kg. On the other hand, ninety‐three samples (93%) had OTA higher than 10 µg/kg (Table 1).

The naturally occurred ochratoxin (OTA) was detected in poultry feedstuff ingredients (Table 2). Ochratoxin A was determined in corn grains, in samples No. 3, 9, 12, 15, and 16, only out of 20 samples. Their levels ranged from 323 and 450 µg/kg. From sorghum grains, only two samples No. 8 and 17 were contaminated by ochratoxin A in levels of 11.3 and 21.6 µg/kg. Six samples out of 20 soya bean had ochratoxin A with levels varied between 18–39 µg/kg. Ochratoxin A was found in only one sample of barley grains with level 4.4 µg/kg. Ochratoxin A was observed in eight samples of wheat bran out of 20 with concentrations ranged from 4.3 to 8.1 µg/kg. From our results, we found that corn grains were more contaminated than other samples which also had the highest ranges of moisture contents (12.2%–15.4%) (Table 2).

**Table 2:** The moisture contents (%) and ochratoxin A analysis (µg/ kg) of 22 positive samples out of 100 from corn grains, sorghum, soya bean, barley, and wheat bran were recovered from Riyadh, Alhassa, Qassium, and Jeddah areas

Sample No.	Sources	Location	M.C.	OTA	Sample No.	Sources	Location	M.C.	OTA
3	Corn grains	Riyadh	14.1	450	13	Soya bean	Qassium	8.3	39
9	Corn grains	Alhassa	14.5	423	20	Soya bean	Jeddah	10.5	34
12	Corn grains	Qassium	12.2	349	4	Barely	Riyadh	10.1	4.4
15	Corn grains	Qassium	12.5	323	1	Wheat bran	Riyadh	10.3	6.7
16	Corn grains	Jeddah	15.4	429	4	Wheat bran	Riyadh	10.2	5.5
8	Sorghum grains	Alhassa	12.1	11.3	6	Wheat bran	Alhassa	12.3	5.3
17	Sorghum grains	Jeddah	13.2	21.6	9	Wheat bran	Alhassa	12.1	7.8
1	Soya bean	Riyadh	7.4	37	13	Wheat bran	Qassium	11.5	5.2
3	Soya bean	Riyadh	7.3	32	15	Wheat bran	Qassium	11.2	4.3
7	Soya bean	Alhassa	9.3	18	18	Wheat bran	Jeddah	13.2	8.1
10	Soya bean	Alhassa	9.5	35	20	Wheat bran	Jeddah	13.3	6.7

Note

M.C., Moisture content; OTA, Ochratoxin A.

Our results revealed that OTA contamination in mixed poultry feedstuff samples (with a maximum level at 14.8 μg/kg) was less than their ingredients (highest concentration at 450 μg/kg) (Tables 1 and 2).

### 3.3 Detection of ochratoxin A in fungal species were isolated from mixed feedstuff samples

Among isolates that were collected from Riyadh samples, 17 tested isolates of *A*.* niger*, 7 isolates were ochratoxigenic, and their average production was 0.34 µg/ kg, while the two tested isolates of *A*.* ochraceus* were positive with the level of 0.45 µg/ kg (Table 3). From the genus *Penicillium,* 5 species namely *P*.* chrysogenum* (20 isolates), *P*.* commune* (2 isolates), *P*.* purpurascens* (1 isolate), *P*.* purpurogenum* (1 isolate), and *P*.* variabile* (1 isolate) produced low levels of ochratoxin A ranged from 0.01 to 0.03 µg/ kg. *P*.* verrucosum* isolate showed a high level (2.5 µg/ kg). (Table 3).

**Table 3:** Ochratoxin A were produced by fungal species which were isolated from mixed and ingredients poultry feedstuff samples that were marketed from Riyadh, Alhassa, Qassium, and Jeddah cities

Species	mixed poultry feedstuff								Ingredients poultry feedstuff										Accession numbers
	Riyadh		Alhassa		Qassium		Jeddah		Corn grains		Sorghum		Soyabean		Barley		Wheat bran		
	PS (TS)	OTA	PS (TS)	OTA	PS (TS)	OTA	PS (TS)	OTA	PS (TS)	OTA	PS (TS)	OTA	PS (TS)	OTA	PS (TS)	OTA	PS (TS)	OTA	
*Aspergillus niger*	7(17)	0.34	8(12)	0.21	9(9)	0.36	10(15)	1.1	6 (9)	1.3	10(19)	0.63	2(3)	0.75	6(17)	0.41	2(2)	0.50	HG964327
*A*.* ochraceus*	2(2)	0.45	0	0	1(1)	0.45	4(4)	0.61	2 (2)	0.91	1(1)	0.7	1(1)	0.85	0	0	3(3)	0.68	HG964328
*Penicillium chrysogenum*	5(20)	0.02	4(13)	0.03	6(6)	0.03	8(19)	0.02	4(10)	0.23	5(17)	0.05	1(17)	0.06	5(17)	0.05	5(10)	0.04	HG964285
*P*.* commune*	1(2)	0.03	0	0	1(1)	0.04	2(2)	0.04	0	0	1(2)	0.04	1(1)	0.04	0	0	0	0	HG964287
*P*.* purpurascens*	1(1)	0.02	1(1)	0.03	0	0	1(1)	0.04	0	0	0	0	0	0	0	0	0	0	HG964299
*P*.* purpurogenum*	1(2)	0.01	2(2)	0.02	0	0	1(2)	0.01	2(2)	0.05	1(1)	0.06	0	0	0	0	0	0	HG964300
*P*.* variabile*	1(2)	0.03	1(1)	0.06	1(1)	0.07	3(3)	0.03	1(1)	0.05	1(1)	0.07	0	0	0	0	0	0	HG964302
*P*.* verrucosum*	1(1)	2.5	0	0	1(1)	4.5	1(1)	5.5	0	0	0	0	0	0	0	0	0	0	HG964303
*P*.* spinulosum*	0	0	0	0	0	0	1(1)	0.01	0	0	0	0	0	0	0	0	0	0	HG964304
*P*.* viridicatum*	0	0	0	0	0	0	1(1)	0.04	0	0	2(2)	5.9	0	0	0	0	9(13)	0.9	HG964305

Note

OTA, Ochratoxin A; PS, positive strains, TS, tested strains.

From isolates were collected from Alhassa, eight out of 12 isolates of *A*.* niger* were ochrtaoxigenic and the average toxin level 0.21 µg/ kg. From the genus *Penicillium*, *P*.* chrysogenum* (4 isolates), *P*.* purpurascens* (1 isolate), *P*.* purpurogenum* (2 isolates), and *P*.* variabile* (1 isolate) were ochratoxigenic and their production ranged from 0.02 to 0.06 µg/ kg (Table 3).

In fungal species were isolated from Qassium samples, all *A*.* niger* isolates (9) were ochratoxin A producers with average level = 0.36 µg/ kg. *A*.* ochraceus* represented by a single isolate produced OTA with 0.45 µg/ kg. Among *Penicillium* species, *P*.* verrucosum* represented by a single isolate showed the highest level of OTA production (4.5 µg/ kg) as shown in Table (3).

From samples were collected from Jeddah, *A*.* niger* (10 isolates) and *A*.* ochraceus* (4 isolates) were OTA producers, their production levels ranged from 1.1 to 0.61 µg/ kg, respectively (Table 3). Among 18 isolates of different *Penicillium* species, only a single isolate of *P*.* verrucosum* was able to produce OTA with 5.5 µg/ kg, while the rest of *Penicillium* isolates produced lower levels of OTA.

### 3.4 Ochratoxin A detection in fungal species were isolated from feedstuff ingredients samples

Six out of nine *A*.* niger* isolates*,* 2 isolates of *A*.* ochraceus,* 4 out of ten isolates of *P*.* chrysogenum,* 2 isolates of *P*.* purpurogenum,* and a single isolate of *P*.* variable* were isolated from corn grains which were collected from the four areas in Saudi Arabia were OTA producers; their production levels ranged from 0.05 to 1.3 µg/ kg (Table 3).

The production levels of OTA with fungal strains that were isolated from sorghum grains were as follows: ten isolates of *A*.* niger* produced 0.63 µg/ kg, while *A*.* ochraceus* produced 0.7 µg/ kg. Two isolates of *P*.* viridicatum* showed the highest amounts of OTA 5.9 µg/ kg, while the other *Penicillium* isolates that belonged to the other penicilli exhibited a lower amount of OTA (Table 3).

The production levels of OTA with *A*.* niger* (2 isolates) that were isolated from soya bean grains ranged from 0.75 µg/ kg, while *A*.* ochraceus* (1 isolate) produced 0.85 µg/ kg. Eighteen isolates of different *Penicillium* species, namely *P*.* chrysogenum* (1 isolate) and *P*.* commune* (1 isolate) were able to produce OTA and their production levels were shown in table (3).

The ochratoxin A potentials of thirty‐four isolates were collected from barley grains: *A*.* niger* (17 isolates) and *P*.* chrysogenum* (17 isolates). Six isolates of *A*.* niger* were OTA producers, and the production levels were 0.41 µg/ kg, while 5 isolates of *P*.* chrysogenum* were OTA producers with low amounts 0.05 µg/ kg as shown in Table (3).

Ochratoxin A potentials of *A*.* niger* (2 isolates), *A*.* ochraceus* (3 isolates), *P*.* chrysogenum* (10 isolates), and *P*.* viridicatum* (13 isolates) were detected from wheat bran samples (Table 3). All isolates of *A*.* niger* and *A*.* ochraceus* were OTA producers, and the production levels ranged from 0.50 to 0.68, while 5 out of 10 isolates of *P*.* chrysogenum* were OTA producers with low amount (0.04 µg/ kg). Nine out of 13 isolates of *P*.* viridicatum* were OTA producers, and their average production levels range 0.9 µg/ kg.

### 3.5 Survey of *pks* genes (ochratoxin biosynthetic genes)

**Figure 3:**
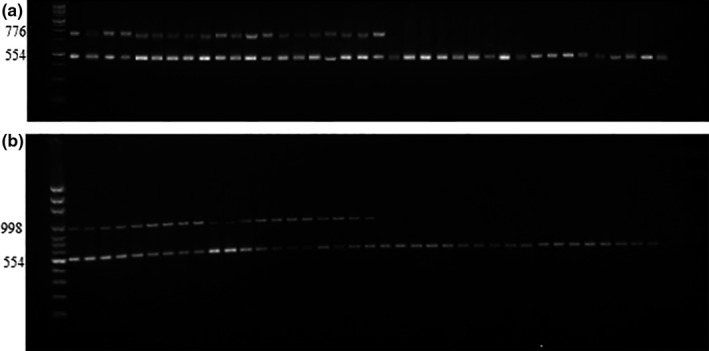
Association between OTA production capability of each strain and presence of *pks* genes, as demonstrated by PCR performed with two primer pairs, PKS15KS (a) and PKS15C‐MeT (b). PCR detected a 776 bp band and a 998 bp band, respectively, in the ochratoxin producing strains only. The 554 bp‐amplicon corresponding to the βt2 region of the β‐tubulin gene confirms the presence of PCR‐compatible DNA in all strains

One hundred and three isolates of *Aspergillus niger* isolates were collected from Riyadh, Alhassa, Qassium, and Jeddah areas were tested for their ochratoxin potentials and absence or presence of *PKs* genes (ochratoxin biosynthetic genes) as shown in Figure (3). Out of 103 isolates, only 63 isolates were ochratoxigenic. All those ochratoxigenic isolates showed the presence of *pks* genes by using PKS15C‐MeT and PKS15KS primer pairs. The ochratoxigenic isolates showed DNA amplicons sizes 998 and 776 bp and the βt2 region of the β‐tubulin gene formed 554 bp‐amplicon which confirmed the presence of PCR‐compatible DNA in all strains. (Figure 3).

### 3.6 Molecular detection of *Aspergillus niger* in mixed poultry foodstuff samples and their ingredients

**Figure 4:**
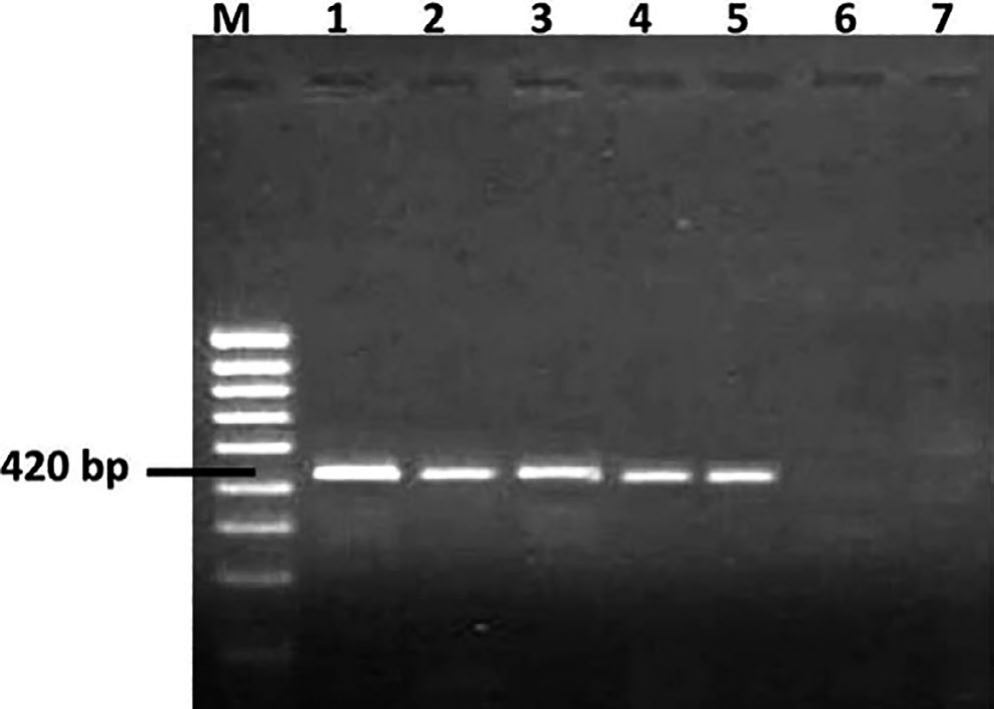
Agarose gel electrophoresis of PCR products of DNA fragments specific for *Aspergillus niger* using primer pairs ITS1 and NIG. Lane 1: Positive control; Lanes 2–5: DNA from samples collected from Riyadh, Alhassa, Qassium, and Jeddah. Lane 6 sample (free *Aspergillus niger*) and lane 7 negative control. M: DNA marker

All collected samples of mixed poultry foodstuff ready for feeding (100 samples) and their ingredients (100 samples) were subjected to enrichment techniques to isolate the total genomic DNA of contaminated fungal species. The collected DNA samples were amplified by ITS1 and NIG primers for detecting the presence of *Aspergillus niger* in the tested samples. The most heavily contaminated samples were chosen and also some of the free *A*.* niger* samples. The contaminated samples amplifiedPCR products at 420 bp, this result indicating the presence of the tested fungus. While no amplicon (PCR product) was scored for free *A*.* niger* samples (Figure 4).

#### 3.6.1 DISCUSSION

Two genera and 10 species were isolated from 200 samples of mixed poultry feedstuff and their ingredients which were collected from four regions in Saudi Arabia. *Penicillium chrysogenum*was the predominant species. *A*.* niger* was the second dominant species in its account and frequency; 3,257.2 & 1920.7 CFU/g and 53% & 50%, respectively, in both mixed poultry feedstuff samples and their ingredients. This result has been in agreement with Kana et al. (2013) who isolated *A*.* flavus*, *A*.* niger*, *A*.* oryzae*, *F*.* solani*, *F*.* verticilloides*, *Penicillium* spp., and *Rhizopus* spp. that the most representative fungi from 202 poultry feed and their raw ingredients which were gathered from different agro‐ecological zones of Cameroon.

Moisture content was an effect on the account of fungi isolated, and we observed that the highest moisture content of mixed poultry feedstuff samples was in Jeddah city (11.5%), which also had the highest account of fungi 3,181 CFU/g. Gherbawy, Shebany, and Alharthy (2019) reported that the highest moisture content was found in poultry feedstuff sample (8.4%) than the other animal feedstuff sample (3.2%). Krnjaja et al. (2014) reported that the moisture content is an important indicator for the contamination of poultry feed samples with fungi, which had moisture contents ranged from 8.04% to 12.67%.

The contamination of agriculture commodities used in the preparation of poultry feed with toxigenic fungi may lead to mycotoxin increase reaching harmful levels for farm animals and consequently for humans. Hence, poultry feed is frequently contaminated by mycotoxins, for that reason, poultry could be subject to mycotoxicoses (Smith, Solomons, Lewis, & Anderson, 1995). All mixed poultry feedstuff samples were contaminated with ochratoxin (OTA) which were collected from four regions in Saudi Arabia. Our results showed that sample of mixed poultry feedstuff No. 100 which was collected from Jeddah appeared the highest level of ochratoxin A (14.8 µg/kg). And also, ninety‐three percent of samples (93%) had OTA higher than 10 µg/kg. Schiavone et al. (2008) recorded that all the tested poultry feed samples were contaminated with OTA varied between 0.04 and 6.50 μg/kg in Italy. Bokhari (2010) found that contamination of compound feeds with aflatoxin and ochratoxin A was ranged from 1 to 6.4 ppb and 1 to 4.7 ppb, respectively. Sifou et al. (2016) reported that OTA was found in 19 (30.6%) out of total tested samples. OTA levels ranged from 0.24 to 26.8 ng/g. The average levels of OTA were 0.8, 9.4, and 11.3 ng/g in samples collected from Salé, Témara, and Rabat, respectively.

In our results, corn grains samples were highly contaminated with ochratoxin A. In Kuwait, Beg et al. (2006) detected ochratoxin A with quantitative enzyme‐linked immunosorbent assay. They reported that the average levels of ochratoxin A ranged from 4.6 to 9.6 ppb, in various commodities and prepared feed samples. Their study revealed the coexistence of determined mycotoxins, although their concentrations, in general, were found to be lower than the permissible levels, wherever defined, for the poultry feed. In Saudi Arabia, the incidence of ochratoxin A in samples of natural feed ingredients was found to be ranged from 1 to 44 ppb (Bokhari, 2010). Fareed, Khan, Anjum, and Ahmed (2014) found that 86.6% of corn samples were contaminated with OTA. The average contamination level and maximum level of OTA in corn were 60 and 97.5 μg/kg, respectively. Our paper showed that OTA contamination in mixed poultry feedstuff samples was less than their ingredients. This has been in agreement with Fareed et al. (2014) who reported that the incidence of OTA in finished feed was 29.17%. The average and maximum contamination levels of OTA were 7.11 and 23 μg/kg, respectively; however, these levels were less than that of feed ingredients (70.7 μg/kg).

From our findings, ochratoxin A production with fungal species which were gathered from mixed feedstuff samples collected from Riyadh, Alhassa, Qassium, and Jeddah cities were highly by *P*.* verrucosum* (5.5 μg/kg) and *A*.* niger* (1.1 μg/kg). Saleemi, Khan, and Khan (2010) reported that ochratoxigenic *Aspergilli* isolates in poultry feeds (*A*.* niger* aggregates and *A*.* ochraceous*) varied in OTA producing abilities from 0.0014 to 16.72 μg/g. In Brazilian poultry feeds, ochratoxin production with species *A*.* niger*, *A*.* ochraceous,* and *P*.* verrucosum* ranged from 25 to 120 μg/kg on CYA medium (Rosa et al., 2006).

It was found that isolates of: *P*.* viridicatum* which was isolated from sorghum grains was the highest species producing of ochratoxin A (5.9 μg/kg) followed by *A*.* niger* that was isolated from corn grains produced toxin in average of 1.3 μg/kg. Czerwiecki, Czajkowska, and Witkowska‐Gwiazdowska (2002) and Pardo, Marin, Ramos, and Sanchis (2004) revealed that ochratoxin A is known to be produced mainly by *P*.* verrucosum, P*.* cyclopium, P*.* viridicatum, A*.* versicolor, A*.* glaucus, A*.* flavus, A*.* niger,* and *A*.* ochraceus*. All these species of OTA producers were isolated from the field, marketed, and stored moldy sorghum in Niger State as reported by Makun et al. (2009). *A*.* ochraceus* and *P*.*viridicatum* produce OTA between 0 and 31°C (Miller & Trenholm, 1994). Miller and Trenholm (1994) showed that *Penicillium spp* can grow and elaborate mycotoxins over a broader range of temperatures than *Aspergillus*.

In our result, sixty‐three isolates out of 103 of *A*.* niger* were ochratoxigenic, and all of them showed the presence of *pks* genes using PKS15C‐MeT and PKS15KS primer pairs. This result was in agreement with Ferracin et al. (2012) who reported the ochratoxin production capability of the 119 *A*.* niger* strains and found a positive correlation between the presence of *pks* gene and the ability of these strains to produce ochratoxin.

In this study, the heavily contaminated samples (mixed poultry feedstuff samples and their ingredients) tested for the presence of *A*.* niger* species, PCR products formed at 420 bp, this result indicating the presence of the tested fungus. The noncontaminated samples (free from *A*.* niger*) did not form PCR products with ITS1 and NIG primers. This result similar to the data were recorded by Jedidi et al. (2018) who revealed that discrimination of the two species of the *A*.* niger* aggregate (*A*.* niger* and *A*.* tubingensis*) from the rest of the species included in the *Nigri* section of *Aspergillus* by using ITS1 and NIG primers, which amplified a single fragment of about 420 bp, and they found that all forty‐three *Aspergillus* section *Nigri* strains were amplified by the ITS1/NIG primers. The direct detection of the fungus in the samples providing a useful tool for early detection of *A*.* niger* in poultry foodstuff and other food systems, so it was a rapid and specific protocol without cultured of the samples on fungal medium.

In conclusion, the moisture content is an important factor in the contamination of poultry feedstuff samples with toxigenic fungi which may lead to mycotoxin increase reaching harmful levels for farm animals and consequently for humans, so drying and avoiding humid conditions are necessary to control the fungal contamination during storage. The early detection assay can be an important in research, epidemiology studies, as well as to control industrial quality aiming to reduce the levels of ochratoxins in poultry foodstuff commodities.

## CONFLICT OF INTEREST

The authors declare that they do not have any conflict of interest.

## ETHICAL STATEMENTS

Ethical review: This study does not involve any human or animal testing.
